# Forensic characteristics of 4866 violent injury cases in Sichuan Province, China

**DOI:** 10.1038/s41598-023-28806-7

**Published:** 2023-04-12

**Authors:** Shilin Zhang, Wei Wang, Mengxuan Wei, Yu Luo, Wu Long, Lincong Li, Chunyue Jiang, Tao Zhu, Xia Lin, Bo Jin

**Affiliations:** 1 Institute of Basic Medicine Sciences and Forensic Medicine, North Sichuan Medical College, Nanchong, 637000 People’s Republic of China; 2 School of Forensic Medicine, China Medical University, Shenyang, People’s Republic of China; 3 School of Forensic Medicine, Kunming Medical University, Kunming, People’s Republic of China; 4 Qinghai Provincial Public Security Department, Xining, People’s Republic of China; 5 Department of Preventive Medicine, North Sichuan Medical College, Nanchong, People’s Republic of China; 6 Department of Clinical Medicine, North Sichuan Medicine College, Nanchong, People’s Republic of China; 7 Department of Rehabilitation Medicine, Affiliated Hospital of North Sichuan Medicine College, Nanchong, People’s Republic of China

**Keywords:** Health care, Health occupations, Risk factors, Environmental social sciences

## Abstract

To evaluate the characteristics of violent injury cases in Sichuan Province, China. Overall, 4866 violent injury cases in Sichuan province, China from 2014 to 2017 were included. The injury evaluation report was used to classify and summarize the injury information, case and injury characteristics, and to describe the characteristics for each risk factor. The majority of cases were males (n = 3851, 79.14%), aged 20–60 (n = 3867, 79.47%), and living in rural areas (n = 3094, 65.55%). Many cases occurred in public areas (n = 3351, 74.19%) and in the evening (n = 1005, 29.49%). Passion was the main motive for the violent injuries (n = 2098, 82.11%) and the main types of injuries were those to the brain, face, and auricula (n = 3075, 63.21%). Blunt instruments (n = 2951, 64.86%) were most commonly used to inflict injury, and the injury evaluation determined that the majority of injuries were simple (n = 2669, 54.85%) and slight (n = 1685, 34.63%). For cases resulting from passion and money, blunt instruments were more commonly used, while sharp instruments were more commonly used for injuries resulting from emotion and revenge (*p* < 0.05). Compared with grievous injuries, public and entertainment areas and the use of blunt instruments were risk factors for slight injuries. The use of blunt instruments was a risk factor for simple injuries. The cases of violent injury in the Sichuan Province of China have certain characteristics and causes. In order to reduce the frequency of such cases, corresponding intervention measures should be actively conducted at the identified high risk places, times, and populations.

## Introduction

In forensic justice, the consequences of violent injury cases are often very serious due to the degree of damage suffered by the victim and the violence enacted by the perpetrator. It not only harms the physical and mental health, and accumulation of property of the parties, but also has negative effects on the event stakeholders and bystanders. Therefore, it is particularly important to discuss how to reduce the occurrence of violent injury cases, by studying the characteristics of such cases. This information can then be used by the relevant legislation and law enforcement agencies to devise corresponding intervention measures.

At present, most of the research on violent injury cases abroad focuses on the cases of death caused by violent injury^1,2^. Some of which include prevention of violence types, measurement of injury results, intervention types and the impact of violence on the economy, etc.^3^. The research on violent injury in China mainly comes from the emergency department of hospitals. Due to the influence of the case source^4^, the evidence on factors affecting violent injuries is not comprehensive. Injury evaluation reports have a more detailed record of violent injury cases; they can determine the forensic characteristics of violent injury cases to a certain extent, and provide theoretical support for the trial and prevention of related cases. This study reported on the characteristic of violent injury cases in Sichuan Province of China, conducted statistical classification, and correlation analysis of related factors.

## Results

### Injury information

Of the 4866 included violent injury cases, the male to female ratio was 3.79:1. The majority of cases were 21–60 years of age (n = 3867, 79.47%). The most common type of injury was that to the brain (n = 1784, 36.66%); the other types of injuries experienced can be seen in Table 1. Simple injuries (n = 2669, 54.85%) were the most common injury evaluations (Table 1). After excluding 146 (3%) cases with missing household information, 3094 of the 4720 injured were from rural areas (n = 3094, 65.55%, Fig. 2a).

**Table 1:** Injury cases detected in Sichuan Province, China, from January 2014 to December 2017.

	Male	Female	Total	*P*-values
	N = 3851 (%)	N = 1015 (%)	4866 (%)	
Age group (years)				< 0.001
0–20	456 (11.8)	76 (7.5)	532 (10.9)	
20–40	1717 (44.6)	361 (35.6)	2078 (42.7)	
41–60	1334 (34.6)	455 (44.8)	1789 (36.8)	
> 60	344 (8.9)	123 (12.2)	467 (9.6)	
Total	3851 (100.0)	1015 (100.0)	4866 (100.0)	
Season of injury				0.223
Spring (February–April)	1084 (28.1)	299 (29.5)	1383 (28.4)	
Summer (May–July)	1013 (26.3)	290 (28.6)	1303 (26.8)	
Autumn (August–October)	832 (21.6)	205 (20.2)	1037 (21.3)	
Winter (November–January)	922 (23.9)	221 (21.8)	1143 (23.5)	
Total	3851 (100.0)	1015 (100.0)	4866 (100.0)	
Type of injury				0.100
Brain	1422 (36.9)	362 (35.7)	1784 (36.7)	
Face and auricular	996 (25.9)	295 (29.1)	1291 (26.5)	
Neck	82 (2.1)	19 (1.9)	101 (20.8)	
Chest	410 (10.6)	105 (10.3)	515 (10.6)	
Abdomen, pelvis and Perineum	203 (5.3)	36 (3.5)	239 (4.9)	
Spine and spinal cord	95 (2.5)	35 (3.4)	130 (2.7)	
Limbs	570 (14.8)	142 (14.0)	712 (14.6)	
Body surface and other	73 (1.9)	21 (2.1)	94 (1.9)	
Total	3851 (100.0)	1015 (100.0)	4866 (100.0)	
Injury evaluation				< 0.001
Grievous	423 (11.0)	89 (8.8)	512 (10.5)	
Simple	2160 (56.1)	509 (50.1)	2669 (54.8)	
Slight	1268 (32.9)	417 (41.1)	1685 (34.6)	
Total	3851 (100.0)	1015 (100.0)	4866 (100.0)	

### Case characteristics

#### Time

**Figure 1:**
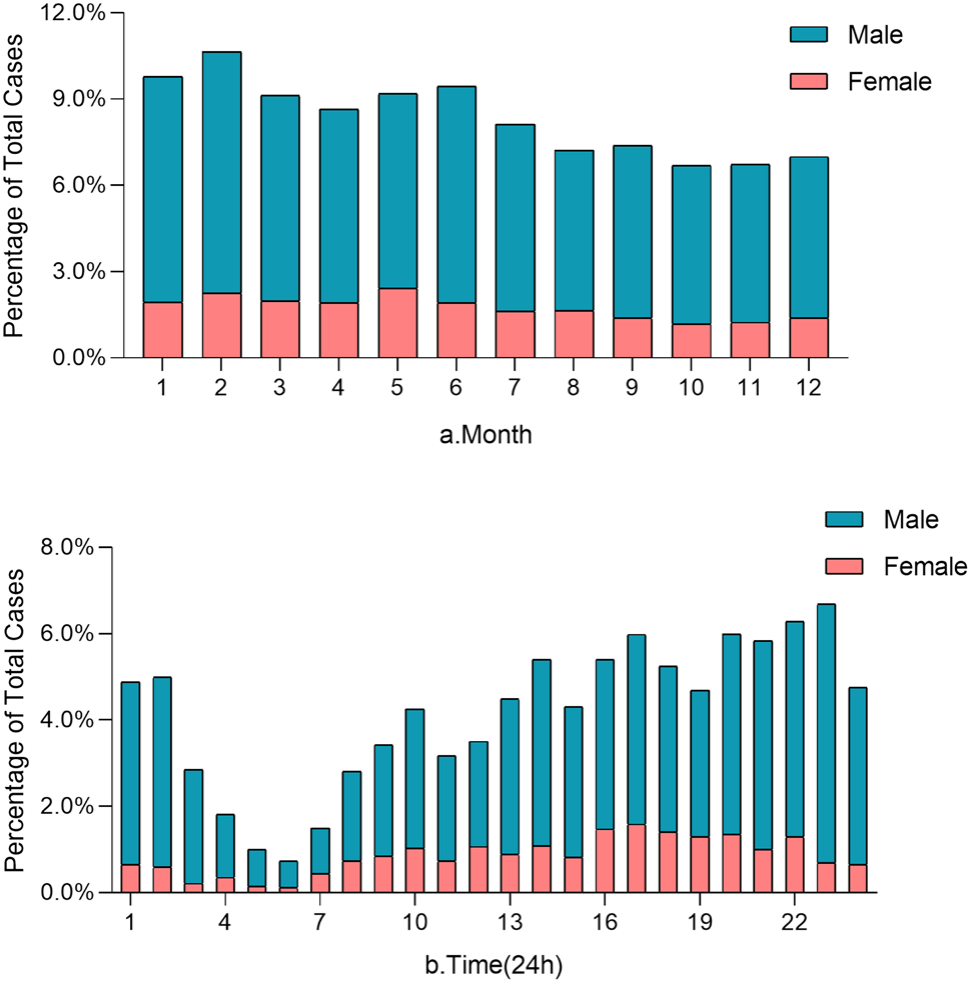
Month and time distribution of violent injury cases. (**a**) The monthly distribution of 4866 intentional injury cases. (**b**) The specific time distribution of 3506 violent injury cases from 0 to 24 h.

The majority of violent injury cases occurred in the spring (n = 1383, 28.42%, Table 1). In February, there was the greatest number of violent injuries (n = 518, 10.65%, Fig. 1 and Supplementary File Table 1). Overall, 700 (14.39%), 658 (13.48%), 718 (14.76%), 706 (14.51%), 664 (13.65%), 710 (14.59%), and 712 (14.63%) cases occurred from Monday to Sunday, respectively.

The time at which the injury was inflicted was clearly documented in 3408 (70.04%) cases; 218 (6.40%), 517 (15.17%), 456 (13.38%), 714 (20.95%), 1005 (29.49%), and 498 (14.61%) cases occurred before dawn, in the forenoon, at noon, in the afternoon, at night, and at midnight, respectively. The distribution of cases from 0 to 24 h is shown in Fig. 1b and Supplementary File Table 1.

#### Place

**Figure 2:**
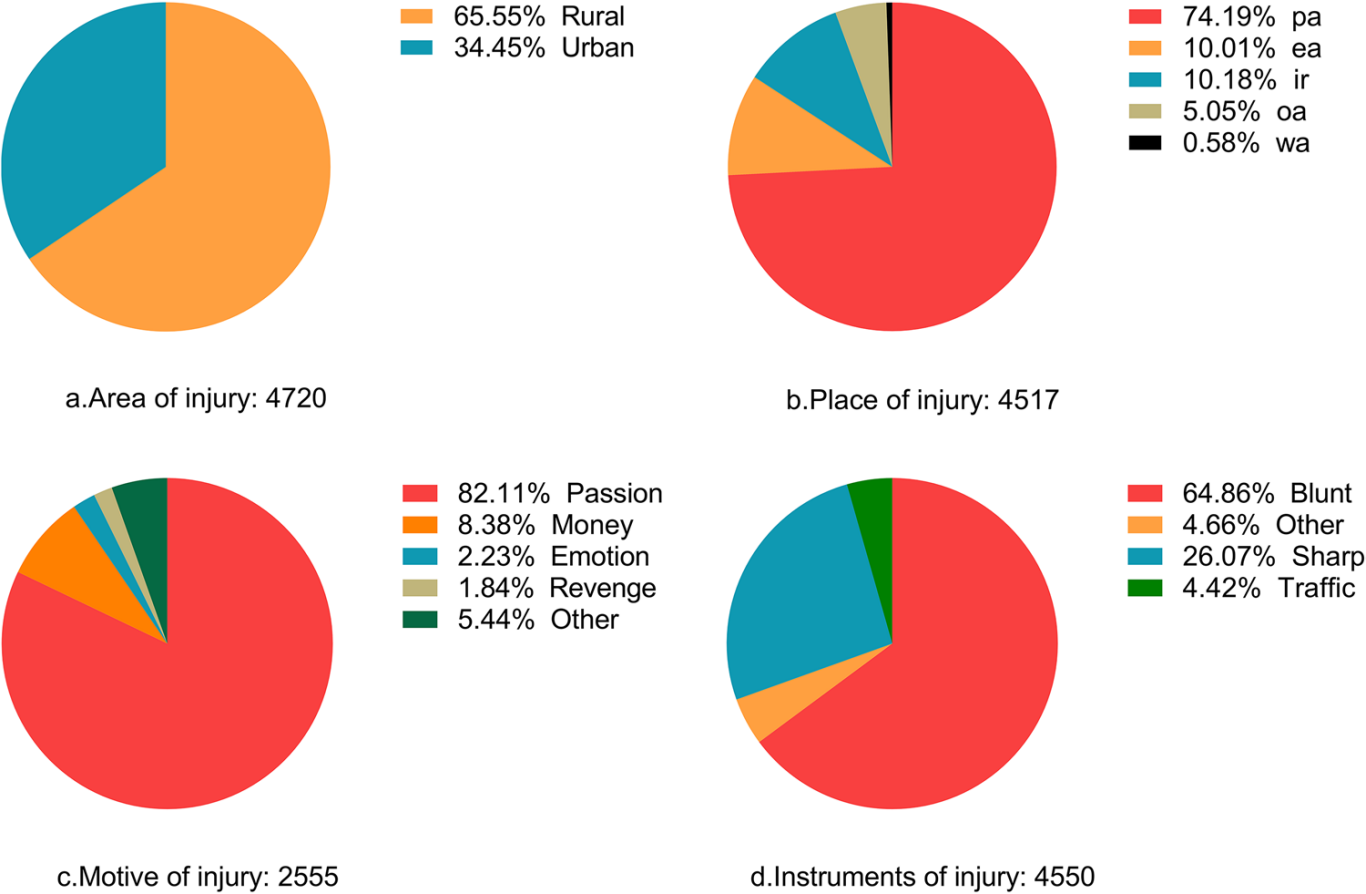
Distribution of (**a**) area and (**b**) place of injury, (**c**) motive for injury, and (**d**) instruments used to inflict injury. Abbreviations: pa, public area; ea, entertainment area; ir, individual residence; wa, wild area; oa, others.

After the exclusion of 349 (7.17%) cases with invalid data, among the remaining 4517 cases, 3351 (74.19%), 452 (10.01%), 460 (10.18%), 26 (0.58%), and 228 (5.04%) occurred in public and entertainment areas, in personal residence, in wild and other areas, respectively (Fig. 2b).

#### Motive

Excluding 2311 (47.50%) cases with invalid data, among the remaining 2555 cases, passion, money, emotion, revenge, and other motives (including family and medical disputes, and accidental injury) were the cause of 2098 (82.11%), 214 (8.38%), 57 (2.23%), 47 (1.84%), 139 (5.44%) cases, respectively (Fig. 2c).

### Instruments used to inflict injury

Excluding 316 cases with invalid data, of the remaining 4550 cases, blunt instruments, sharp instruments, traffic mainly caused by non-motor vehicles, and other instruments were used to inflict injury in 2951 (64.86%), 1186 (26.07%), 201 (4.42%), and 212 (4.66%) cases, respectively (Fig. 2d). Of the blunt instruments, 2446 (53.76%) were the fist and foot, while 1109 (24.37%) of sharp instruments were knives (Supplementary File Table 2).

### Correlation analysis for place of and motive for injury and instruments used to inflict injury

There were 4137 cases of injury caused by blunt and sharp instruments, accounting for 90.93% of the 4550 cases; only 413 (8.51%) cases resulted from the use of other instruments. Therefore, this study mainly analyzed the correlation between blunt and sharp instruments (Table 2).

**Table 2:** Places of and motive for injury stratified by the type of instrument used to inflict injury.

	Blunt	Sharp	Total	*P*-values
Places	N = 2830 (percentage of total)	N = 1136 (percentage of total)	3966	< 0.001
Public area	2136 (74.1)	745 (25.9)	2881	
Entertainment area	250 (58.1)	180 (41.9)	430	
Personal residence	275 (65.6)	144 (34.4)	419	
Other	154 (72.6)	58 (27.4)	212	
Wild area	15 (62.5)	9 (37.5)	24	
Motive	N = 1791 (percentage of total)	N = 590 (percentage of total)	2381	< 0.001
Passion	1544 (77.0)	460 (23.0)	2004	
Money	150 (73.9)	53 (26.1)	203	
Emotion	24 (45.3)	29 (54.7)	53	
Revenge	23 (48.9)	24 (51.1)	47	
Other	50 (67.6)	24 (32.4)	74	

In 3966 cases the instruments used to inflict injury and the place in which the injury took place were clearly recorded. The number of cases caused by blunt instrument was significantly higher than that caused by sharp instruments in all places (*P* < 0.05).

Overall, 2381 cases had a clear record of the instrument used to inflict injury and motive for injury. In cases in which passion and money were the main motive for inflicting injury, the number of cases in which blunt instruments were used was significantly higher than those in which sharp instruments were used. For cases in which the motivation for inflicting injury was emotion and revenge, the number of cases in which sharp instruments were used was significantly higher than those in which blunt instruments were used (*P* < 0.05, Table 2).

There were 2490 cases in which the place and motive for the violent injury were clearly document. There were significant differences in the motive for infecting injury based on the place in which the injury occurred (*P* < 0.001). Passion was the most common motive for injury in each place (Table 3).

**Table 3:** Places in which injuries occurred stratified by motives for inflicting injuries.

	Passion N = 2045 (percentage of total)	Money N = 210 (percentage of total)	Other N = 132 (percentage of total)	Emotion N = 57 (percentage of total)	Revenge N = 46 (percentage of total)	Total N = 2490
Public area	1509 (82.4)	144 (7.9)	117 (6.4)	28 (1.5)	34 (1.9)	1832
Personal-residence	215 (76.8)	33 (11.8)	6 (2.1)	23 (8.2)	3 (1.1)	280
Entertainment-area	223 (85.1)	23 (8.8)	4 (1.5)	4 (1.5)	8 (3.1)	262
Other	86 (86.9)	5 (5.1)	5 (5.1)	2 (2.0)	1 (1.0)	99
Wild area	12 (70.6)	5 (29.4)	0 (0.0)	0 (0.0)	0 (0.0)	17

### Risk factor analysis for injury evaluation

After excluding cases in which there were incomplete records, 2016 cases were included in the multivariate logistic regression analysis (Table 4). Compared with cases of grievous injuries, males, the use of sharp instruments, traffic as a causative agent, and emotion were the protection factors for slight injuries. Conversely, public and entertainment areas and blunt instrument were risk factors for slight injuries. Compared with grievous injuries, occurrence before dawn was a protective factor and the use of a blunt instrument was a risk factor for simple injuries.

**Table 4:** Multivariate binary logistic regression model for injury evaluation using only cases with complete information for model variables (N = 2016) (control = grievous injury).

Variable	Slight			Simple		
	Beta	Odds ratio	95% CI	Beta	Odds ratio	95% CI
Sex (control = female)						
Male	− 0.548*	0.578	0.365–0.914	− 0.65	0.937	0.601–1.460
Place (control = other)						
Public area	2.219**	8.409	2.528–27.970	− 1.00	0.905	0.387–2.115
Entertainment area	1.635*	5.127	1.376–19.112	0.376	1.457	0.551–3.852
Instruments (control = other)						
Blunt	1.169*	3.219	1.196–8.659	1.198*	3.313	1.259–8.719
Sharp	− 1.079*	0.340	0.125–0.929	− 0.027	0.974	0.370–2.560
Traffic accident	− 2.054**	0.128	0.032–0.518	− 0.242	0.785	0.223–2.762
Motive (control = other)						
Emotion	− 1.669*	0.188	0.046–0.768	0.242	1.274	0.427–3.803
Time (control = midnight)						
Before dawn	− 0.371	0.690	0.332–1.434	− 0.793*	0.453	0.228–0.899

*CI* confidence interval.

*P < 0.05, **P < 0.01.

## Discussion

In this study, the frequency of injured males (79.14%) was greater than that of females, as was found in many domestic and foreign research studies^4,5^. This may be a result of the wide range of activities males perform related to life and work; males’ excitability and tendency to use direct and violent means to solve conflicts, and the physiological differences between males and females may also affect the differences in violence based on sex^6^. One study suggested that^7^ men are more prone to violence than women. Additionally, the majority (79.47%) of the injured were 21–60 years of age, which was similar to the findings from another study^8,9^. People in this age range might be stronger than children and adolescents and their temperament might be more straightforward and impatient. The majority of injuries (65.55%) occurred in rural households; this was equivalent to the proportion of rural household registration (approximately 60% on average) issued by the China Statistics Bureau in 2014–2017. The cultural quality and legal awareness of rural residents is poor.

We found violent injury cases at different periods of time. The number of violent injury cases was the highest in February (spring), followed by summer. These findings can be explained by the following. First, February coincides with the Spring Festival in China, thus, there is a sharp increase in the flow of people, which increases the risk of violent injury cases. Second, some studies have shown that^10^ serotonin is related to violence. The number of suicide cases in the northern hemisphere peaks in the summer and spring^11^, because serotonin in the human body is related to sunlight. Some studies have found that^12^ the serotonin levels in the human body reach their peak in summer; China is located in the northern hemisphere and thus the sunshine is the greatest in the spring and summer. The increase in serotonin levels in the human body over these periods lead to more violent injuries. Thirdly, alcohol is a key factor that affects the occurrence of violence. Hot summer weather increases the probability of drinking, which leads to the increase of violent injury cases^13^. Conversely, some studies have found that^14^ the incidence of violence among children in February is relatively low. This may be due to the fact that during the winter vacation in February, schools are closed and there are fewer opportunities to gather, reducing the probability of injury. Over the course of a week, there were cases of violent injury, but the number of cases did not drastically differ.

Based on the findings in this study, there were more violent injury cases in the evening; this is consistent with the findings from previous studies. Schuurman et al.^15^ believe that there is obvious time compliance in injury cases; they found that more intentional violent injuries occur at night, while unintentional violent injuries peak later in the afternoon. This may be due to the fact that most people are working or studying during the day. At night, people are mostly in a state of leisure and entertainment; the probability of contact with strangers' and friction increases, and the field of vision at night is more limited than during the day. Thus, it is not as easy to attract others attention, thus increasing the probability of violent injury. Other studies have found that^16^ this may be related to the fact that after people experience a day's work, people feel fatigued in the evening and therefore are more likely to have an altered mood. Additionally, a study of the mouse biological clock found that^17^ the stimulation of white light at night can enhance the excitability of the adrenocortical sympathetic nerve, thus increasing the cases of violent injury at night.

Injury was more likely to occur in specific places in this study; the majority of cases (74.19%) occurred in public areas, followed by entertainment areas; only two cases occurred in the wild areas. Similar findings have been documented in studies conducted in China and abroad^18,19^. The population density is greater in public areas, which makes it more likely for violent injuries to occur. Violent injuries leading to death often occur in remote places such as the wild or at one’s personal residence^20^; this may be a result of the small number of people in such locations, the lack of public attention, and the ease with which the perpetrator can escape. In this study, we found that the majority (82.11%) of violent injury cases were due to passion. This is likely due to trivial quarrels or family conflicts, which might have led to impulsive actions and emotions and a lack of rational thinking. These findings reflect the low level of psychological wellbeing, poor self-control and ability to deal with contradictions, and limited legal awareness.

We found that blunt instruments were most commonly (64.86%) used to inflict injury. There were significant differences in the use of blunt and sharp instruments based on places of and the motive for injury. In public areas, blunt instruments were more commonly used, and the majority of the motives were passion. This may be due to the fact that blunt instruments, including the fists, feet, bricks, wine bottles, and sticks, are easy to obtain They are easily accessible in daily life, and are available in all public places. Therefore, it is necessary to take appropriate restrictive measures to prevent such injuries. For example, it is strictly prohibited to carry sticks, bricks and stones in public areas in China; it is thus necessary to ensure that such instruments are restricted in public areas. In entertainment areas, empty wine bottles should be recovered and stored in a timely manner. Our findings are similar to those from some previous studies. Hwa et al.^21^ analyzed murders among Taiwanese children aged 0–17 from 2001 to 2010 and found that blunt instrument were the most commonly used instruments to inflict injury. Ben et al.^22^ found that the most common killing methods were sharp weapons and blunt attacks. Other methods are more complex to use, not easy to obtain and carry, and require certain conditions, thus, there are fewer cases resulting from such methods. These findings differ from those in some countries in which firearms were the main cause of violent injuries^23,24^. In China there are strict gun control systems and thus injuries resulting from the use of firearms are not common. Additionally, some cases were caused are a result of traffic injuries, mainly by non-motor vehicles, indicating that there needs to be strengthened management of the use of non-motor vehicles and the formulation of improved laws. There may be a correlation between the choice of instruments used for inflicting injury and the motivation of the perpetrator. In the cases of emotion and revenge, the instruments used to inflict injury were mostly sharp, while in the cases of passion or money, the instruments used to inflict injury were mainly blunt.

We found that the majority of injuries were to the brain, face, and auricula and limbs, which are mostly protruding and easy to access. When getting attached, it is likely that the individual will instinctively curl up, thus the chest and the abdomen are protected, while the brain and limbs are exposed and thus easy to injury.

In all cases, the injury evaluation showed slight injury (34.63%) and simple injury (54.85%); these findings are similar to those from some previous studies^8,14^. Upon the analysis of risk factors for different injury evaluations, it was found that males were more likely to be seriously injured in violent injury cases. This may be due to the previously discussed factors. Sharp instruments are easy to penetrate objects and thus are easy to cause damage, especially to the tissues and organs, which can lead to fatality. Thus, they are more likely to be associated with a grievous injury. Cases in which emotions were the motive for injury were more likely to be grievous. Emotion can easily cause anger among suspects, and result in ignoring the consequences of inflicting injury; these findings are similar to those reported in previous studies^25^. At present, public and entertainment areas are the most crowded places, so the probability of violent injuries there is great.

At present, the research on injury cases in China mainly focuses on patients treated in the emergency department of hospitals, and most of them are non-violent injury cases. Violent injury is a risk factor that causes personal health and social problems and also involves the determination of criminal responsibility. However, violent injury cases are rarely reported in China. Long et al.^18^ analyzed the characteristics of 1340 violent injury cases in southwest China. The results showed that most of the injured were young adults aged 20–50 years (65.2%), male (82.3%), rural household registration (62.8%). Most of the injuries were blunt instruments (54.6%), and the cases mainly occurred in public places (59.0%). January (11.3%), February (13.1%), March (11.6%) and the time from 22:00 pm to 01:00 am (23.2%) were a high incidence period. The injuries mainly are craniofacial injuries, which is different from the result concluded from the thesis. These differences might be related to the number and the source of the samples. These violent injury cases mainly collected from Sichuan Province, China. The research will be strive to expanded the scope and numbers of samples, which means all province will be included. As a result, the research can further compare the differences and similarities between the forensic characteristics of violent sexual injury cases in different provinces. At the same time, according to the characteristics of violent sexual injury cases, relevant preventive measures can be provided to reduce the occurrence of violent sexual injury cases, such as reasonable arrangement of public security patrols at different places and times and publicity of the consequences of violent sexual injury cases.

### Limitations

This study was subject to several limitations. First, only some relevant confounding factors were evaluated; no information on education level, family structure, socioeconomic status, or health conditions were evaluated. Second, the relevant information of suspects was been comprehensively analyzed. The number of cases included in this study was nearly 5000, but after classification based on different factors, the number of cases in some groups was small due to the need to exclude those with missing data. Thus further studies including larger numbers of patients are needed in order to prove the validity of our findings. Moreover, in this study, there were some cases in which the place, motive for, and time of the violent injury were not recorded. This likely had an impact on multivariate binary logistic regression model for injury evaluation. In future, this information should be described in the injury evaluation report and evaluated.

## Conclusions

In conclusion, this study found that violent injury cases in the Sichuan Province, China were comprised of males, those aged 21–60 years of age, and those registered in rural areas. The cases mainly occurred in spring and summer, with the peak occurring in the evening. Cases of violent injury were the most common in public areas, and the main motive for inflicting injury was passion. The main injuries were to the brain and face. There were different risk factors for different injury evaluations. This paper summarizes the characteristics of violent injuries; these findings are expected to provide direction for the future trial and reconnaissance of related cases. Additionally, relevant departments can use these findings to actively implement corresponding intervention measures.

## Methods

### Ethical statements

The study is retrospective study. The need for ethics approval and written informed consent were waived by the Institutional Review Board of North Sichuan Medical College. The study was conducted in accordance with the Declaration of Helsinki. And the data used in this study were anonymized before its use. None of the factors involved in the study could be matched to the any specific individual. All methods were carried out in accordance with relevant guidelines and regulations.

### Study design and participants

In total, 4866 violent injury cases in Sichuan Province, China who underwent injury evaluation at the Forensic Medicine Service Center from January 1, 2014 to December 31, 2017, were randomly selected for inclusion. If the type of injury was unknown or the injury evaluation results were not available and Violent death cases, then the case was excluded from the study.

### Variables and categorization

Based on the injury evaluations, information on demographic (age, gender, and area), case (time, place, and motive for injury), and injury (type of injury, injury evaluation, and instruments used to inflict injury) characteristics were evaluated. The correlation between the instruments used to inflict injury, the place, and the motive for inducing injury and the risk factors based on five factors from the injury evaluation were evaluated.

Time over 24 h was divided into different categories based on classification standards as follows: 02:00–06:00, before dawn; 06:00–11:00, forenoon; 11:00–14:00, noon; 14:00–18:00, afternoon; 18:00–23:00, evening; and 23:00–02:00, midnight.

The places were divided into public entertainment, and wild areas, personal residences, and others. Public areas were classified according to the regulations on the Administration of Public Place Health issued by the State Council of the People's Republic of China on April 1, 1987. Entertainment areas are different from other public areas, such as conflict prone, emotional, and so on. Therefore, they were separately classified in this study. The motives for inflicting injuries were divided as passion, money, revenge, emotional, and others. The instruments used to inflict injury were divided as blunt and sharp instruments, traffic, and others (poisoning, high and low temperature, and electric current). Based on the injury evaluation report, the injured body parts were documented. If there were independent injuries in multiple different body parts, the body parts with the greatest injuries were to be included. Cases of body surface and other injuries were classified as all skin injuries using the same injury evaluation. This was done because the injuries involved multiple parts of the body and did not exist in each body part independently. The injury evaluation classified injuries as grievous, simple, and slight according to the injury evaluation report. The degree of injury recorded in the injury evaluation is graded according to the guidance of the Standard for Identification of Human Injury compiled by the Judicial Appraisal Administration of the Ministry of Justice of China. If an injured person had multiple injuries at the same time, the most serious injury evaluation was recorded. In some cases, information on individual characteristics was missing; such cases were excluded from analysis.

### Statistical analysis

Spss25.0 was used for statistical analysis; chi square (χ2) tests were used for correlation analysis and multiple logistic regression analysis was conducted to evaluate the risk factors for injuries. *P*-values < 0.05 were considered statistically significant. Graphpad prism 8 was used for all of the included images.

## Supplementary Information

Supplementary Tables.

## Data Availability

The datasets used and/or analysed during the current study are available from the corresponding author on reasonable request.
