# Supplementary material for: Forensic characteristics of 4866 violent injury cases in Sichuan Province, China
Source: Sci Rep. 2023 Apr 12;13:5959. doi: 10.1038/s41598-023-28806-7 (PMC10097724; doi:10.1038/s41598-023-28806-7)
Supplement: Supplementary file 1 — Supplementary Tables. [file 41598_2023_28806_MOESM1_ESM.docx]

Supplementary File table1: months and hours of injury.

| **Time of injury** | **No. of cases** | **Percentage of total** |
| --- | --- | --- |
| **Months** | 4866 | 100 |
| [January](E:/Program%20Files%20(x86)/Youdao/Dict/8.6.2.0/resultui/html/index.html#/javascript:;) | 476 | 9.78 |
| [February](E:/Program%20Files%20(x86)/Youdao/Dict/8.6.2.0/resultui/html/index.html#/javascript:;) | 518 | 10.65 |
| [March](E:/Program%20Files%20(x86)/Youdao/Dict/8.6.2.0/resultui/html/index.html#/javascript:;) | 444 | 9.12 |
| [April](E:/Program%20Files%20(x86)/Youdao/Dict/8.6.2.0/resultui/html/index.html#/javascript:;) | 421 | 8.65 |
| [May](E:/Program%20Files%20(x86)/Youdao/Dict/8.6.2.0/resultui/html/index.html#/javascript:;) | 447 | 9.19 |
| [June](E:/Program%20Files%20(x86)/Youdao/Dict/8.6.2.0/resultui/html/index.html#/javascript:;) | 460 | 9.45 |
| [July](E:/Program%20Files%20(x86)/Youdao/Dict/8.6.2.0/resultui/html/index.html#/javascript:;) | 396 | 8.14 |
| [August](E:/Program%20Files%20(x86)/Youdao/Dict/8.6.2.0/resultui/html/index.html#/javascript:;) | 352 | 7.23 |
| [September](E:/Program%20Files%20(x86)/Youdao/Dict/8.6.2.0/resultui/html/index.html#/javascript:;) | 359 | 7.38 |
| [October](E:/Program%20Files%20(x86)/Youdao/Dict/8.6.2.0/resultui/html/index.html#/javascript:;) | 326 | 6.70 |
| [November](E:/Program%20Files%20(x86)/Youdao/Dict/8.6.2.0/resultui/html/index.html#/javascript:;) | 327 | 6.72 |
| [December](E:/Program%20Files%20(x86)/Youdao/Dict/8.6.2.0/resultui/html/index.html#/javascript:;) | 340 | 6.99 |
| **Hours** | 3408 | 100 |
| 1 | 166 | 8.51 |
| 2 | 170 | 4.99 |
| 3 | 97 | 2.85 |
| 4 | 62 | 1.82 |
| 5 | 34 | 1.00 |
| 6 | 25 | 0.73 |
| 7 | 51 | 1.50 |
| 8 | 96 | 2.82 |
| 9 | 117 | 3.43 |
| 10 | 145 | 4.25 |
| 11 | 108 | 3.17 |
| 12 | 119 | 3.49 |
| 13 | 153 | 4.49 |
| 14 | 184 | 5.40 |
| 15 | 147 | 4.31 |
| 16 | 184 | 5.40 |
| 17 | 204 | 5.99 |
| 18 | 179 | 5.25 |
| 19 | 160 | 4.69 |
| 20 | 204 | 5.99 |
| 21 | 199 | 5.84 |
| 22 | 214 | 6.28 |
| 23 | 228 | 6.69 |
| 24 | 162 | 1.12 |

Supplementary File [table](E:/Program%20Files%20(x86)/Youdao/Dict/8.6.2.0/resultui/html/index.html#/javascript:;) 2: instruments of injury (excluding not assigned N=316)

| **instruments of injury** | **NO.of cases** | **Percentage of total** |
| --- | --- | --- |
| **Blunt** | 2951 | 64.86 |
| fists and feet | 2446 | 53.76 |
| stick | 167 | 3.67 |
| hammer | 24 | 0.53 |
| hoe | 29 | 0.64 |
| [brick](E:/Program%20Files%20(x86)/Youdao/Dict/8.6.2.0/resultui/html/index.html#/javascript:;) | 82 | 1.80 |
| Other [metalware](E:/Program%20Files%20(x86)/Youdao/Dict/8.6.2.0/resultui/html/index.html#/javascript:;) | 83 | 1.82 |
| glassware | 104 | 2.29 |
| [plastic](E:/Program%20Files%20(x86)/Youdao/Dict/8.6.2.0/resultui/html/index.html#/javascript:;) [products](E:/Program%20Files%20(x86)/Youdao/Dict/8.6.2.0/resultui/html/index.html#/javascript:;) | 16 | 0.35 |
| **Sharp** | 1186 | 26.07 |
| [knife](E:/Program%20Files%20(x86)/Youdao/Dict/8.6.2.0/resultui/html/index.html#/javascript:;) | 1109 | 24.37 |
| scissor | 10 | 0.22 |
| cullet | 39 | 0.86 |
| [tooth](E:/Program%20Files%20(x86)/Youdao/Dict/8.6.2.0/resultui/html/index.html#/javascript:;) | 12 | 0.26 |
| axe | 16 | 0.35 |
| **Traffic** | 201 | 4.42 |
| **Other** | 212 | 4.66 |
| firearms | 18 | 0.40 |
| high or low temperature | 18 | 0.40 |
| elctric current | 4 | 0.09 |
| poisons | 1 | 0.02 |
| Other instruments besides the above | 171 | 3.76 |
| **Total** | 4550 | 100 |
